# Patterns of Dietary Blood Markers Are Related to Frailty Status in the FRAILOMIC Validation Phase

**DOI:** 10.3390/nu15051142

**Published:** 2023-02-24

**Authors:** Thorsten Henning, Bastian Kochlik, Ignacio Ara, Marcela González-Gross, Edoardo Fiorillo, Michele Marongiu, Francesco Cucca, Fernando Rodriguez-Artalejo, Jose Antonio Carnicero Carreño, Leocadio Rodriguez-Mañas, Tilman Grune, Daniela Weber

**Affiliations:** 1 Department of Molecular Toxicology, German Institute of Human Nutrition Potsdam-Rehbruecke (DIfE), 14558 Nuthetal, Germany; 2 Food4Future (F4F), c/o Leibniz Institute of Vegetable and Ornamental Crops (IGZ), Theodor-Echtermeyer-Weg 1, 14979 Grossbeeren, Germany; 3 NutriAct-Competence Cluster Nutrition Research Berlin-Potsdam, 14558 Nuthetal, Germany; 4 Institute of Nutritional Science, University of Potsdam, 14469 Potsdam, Germany; 5 Department of Nutrition and Gerontology, German Institute of Human Nutrition Potsdam-Rehbruecke (DIfE), 14558 Nuthetal, Germany; 6 GENUD Toledo Research Group, Universidad de Castilla-La Mancha, 45071 Toledo, Spain; 7 CIBER of Frailty and Healthy Aging, CIBERFES, Instituto de Salud Carlos III, 28029 Madrid, Spain; 8 ImFINE Research Group, Department of Health and Human Performance, Universidad Politécnica de Madrid, 28040 Madrid, Spain; 9 CIBER on Physiopathology of Obesity and Nutrition, CIBEROBN, Instituto de Salud Carlos III, 28029 Madrid, Spain; 10 Institute for Genetic and Biomedical Research, National Research Council (CNR), 09042 Monserrato, Italy; 11 Department of Biomedical Sciences, University of Sassari, 07100 Sassari, Italy; 12 Department of Preventive Medicine and Public Health, CIBERESP and IMDEA-Food Institute, Universidad Autonoma de Madrid, CEI UAM + CSIC, 28029 Madrid, Spain; 13 Biomedical Research Foundation, Getafe University Hospital, 28905 Getafe, Spain; 14 Division of Geriatrics, Hospital Universitario de Getafe, 28905 Getafe, Spain

**Keywords:** biomarker patterns, frailty, carotenoids, tocopherols, vitamins, FRAILOMIC

## Abstract

The influence of nutritional factors on frailty syndrome is still poorly understood. Thus, we aimed to confirm cross-sectional associations of diet-related blood biomarker patterns with frailty and pre-frailty statuses in 1271 older adults from four European cohorts. Principal component analysis (PCA) was performed based on plasma levels of α-carotene, β-carotene, lycopene, lutein + zeaxanthin, β-cryptoxanthin, α-tocopherol, γ-tocopherol and retinol. Cross-sectional associations between biomarker patterns and frailty status, according to Fried’s frailty criteria, were assessed by using general linear models and multinomial logistic regression models as appropriate with adjustments for the main potential confounders. Robust subjects had higher concentrations of total carotenoids, β-carotene and β-cryptoxanthin than frail and pre-frail subjects and had higher lutein + zeaxanthin concentrations than frail subjects. No associations between 25-Hydroxyvitamin D3 and frailty status were observed. Two distinct biomarker patterns were identified in the PCA results. The principal component 1 (PC1) pattern was characterized by overall higher plasma levels of carotenoids, tocopherols and retinol, and the PC2 pattern was characterized by higher loadings for tocopherols, retinol and lycopene together and lower loadings for other carotenoids. Analyses revealed inverse associations between PC1 and prevalent frailty. Compared to participants in the lowest quartile of PC1, those in the highest quartile were less likely to be frail (odds ratio: 0.45, 95% CI: 0.25–0.80, *p* = 0.006). In addition, those in the highest quartile of PC2 showed higher odds for prevalent frailty (2.48, 1.28–4.80, *p* = 0.007) than those in the lowest quartile. Our findings strengthen the results from the first phase of the FRAILOMIC project, indicating carotenoids are suitable components for future biomarker-based frailty indices.

## 1. Introduction

Current estimates predict that the proportion of people aged 65+ will rise to 16% of the world’s population by 2050, resulting in a total of 1.5 billion people worldwide [1]. With higher life expectancy, there is also an increasing need to stay physically healthy and active even with advanced age. However, as aging progresses, the prevalence of frailty and sarcopenia increases, leading to a higher risk for falls, fractures, hospitalization, loss of life quality and premature death [2,3,4,5]. Physical frailty is a multifactorial medical syndrome characterized by “diminished strength, endurance, and reduced physiologic function that increases an individual’s vulnerability for developing dependency and/or death” [6,7]. 

For the diagnosis of frailty, there is no universal gold standard to date, with the frailty phenotype by Fried et al. and the frailty index by Rockwood et al. being the most commonly used methods [8,9]. The frailty phenotype described by Fried is based on cut-off values for grip strength, walking speed, unintentional weight loss and low physical activity as well as self-reported exhaustion [8]. The frailty index by Rockwood is calculated in relation to a person’s accumulative health deficits [9]. Novel approaches of calculating a person’s frailty index focus on biomarkers and biomarker patterns. A biomarker-based frailty index including inflammatory, hematological, immunological, senescence, genetic and epigenetic markers was suitable for predicting mortality when compared to isolated biomarkers [10]. Additional biomarkers representing muscle protein turnover, oxidative stress or dietary habits might be of complementary use in frailty diagnoses [11]. In this regard, frail patients were characterized by pro-inflammatory and muscle catabolic patterns by using biomarkers for inflammation and muscle protein turnover as well as dietary nutrients [12]. However, further studies validating such biomarker patterns in large-scale populations are still needed. 

The FRAILOMIC Initiative aims to identify and to validate predictive biomarkers that turn frailty into disability [13]. In the first phase of the project, diagnostic biomarkers for predicting the risk of frailty were identified, but they should subsequently be confirmed in independent cohorts within the second project phase. The study cohort of the first FRAILOMIC phase included participants from four European study centers in France, Spain and Italy [13]. Carotenoids, a group of fat-soluble phytochemicals mainly found in green, red, orange and yellow fruits and vegetables were thereby identified as potential markers for frailty in the first phase [14,15]. Frail participants had significantly lower plasma concentrations of carotenoids such as lutein + zeaxanthin, α-carotene, β-carotene, lycopene and β-cryptoxanthin as well as higher levels of oxidative stress than their robust counterparts [14]. No significant association between plasma levels of α-tocopherol and retinol with frailty status was observed [14]. Therefore, in this study, we aimed to confirm the results from the first project phase in four European cohorts independent from the first phase. We further investigated the associations of dietary blood biomarker patterns, representative of high and low intakes of fruits and vegetables, with the prevalence of frailty.

## 2. Materials and Methods

Participants in this study included men and women aged 65+ years from four European cohorts that were part of the FRAILOMIC Initiative. The Toledo Study of Healthy Aging (TSHA), the Study on Nutrition and Cardiovascular Risk in Spain (ENRICA), the SardiNIA project and the study on the prevalence of overweight and obese non-institutionalized Spanish elders (EXERNET). Ethical approval was obtained from respective institutional ethics committees for each individual cohort.

TSHA: The Toledo Study for Healthy Aging was a population-based study conducted in 2488 individuals aged 65 years and older aiming to assess the prevalence of frailty syndrome in the older adult population of Spain [16]. The study included institutionalized and community-dwelling persons from rural and urban settings around Toledo, Spain. Participants’ data were collected from 2006 to 2009 and included information on social support, activities of daily living, comorbidity, physical activity, quality of life, depressive symptoms and cognitive function. Of the 2488 enrolled participants, 1751 underwent physical examination and provided a fasting blood sample (70.4%). 

ENRICA: This was a cross-sectional survey of 11,991 individuals, representative of the non-institutionalized population aged 18 years and older, who were recruited from June 2008 to October 2010 [17]. The study aimed to assess the frequency and distribution of the main components of the natural history of cardiovascular disease in Spain, including food consumption and other behavioral risk factors as well as biological risk factors, early damage of target organs and diagnosed morbidity. Of the 12,880 individuals selected for physical examination and blood sampling (12-h fasting), 11,191 completed both (86.9%), of which 2439 participants were aged ≥65 years. A subgroup of 498 of those individuals were included in our study. 

EXERNET: The cohort was evaluated within the framework of the elderly EXERNET multi-center study performed on a representative sample of non-institutionalized Spanish seniors aged 65–92 years. The EXERNET study is an ongoing study aiming to provide an update on the prevalence of overweight and obese elders as well as fitness in a representative sample of the non-institutionalized Spanish elderly population. Participants were enrolled in six different locations across Spain (Aragón, Castilla La Mancha, Castilla León, Madrid, Extremadura and Canarias). A total of 3136 subjects underwent physical examination for assessment of anthropometric characteristics, physical activity and fitness and general health data by using validated methods (overall participation rate, 87.1%) [18,19]. Baseline data were collected between June 2008 and November 2009. A subgroup of 431 participants with fasting blood samples from the 2017 data collection wave were included in our study. 

SardiNIA: The SardiNIA cohort consisted of 6148 individuals aged 14–102 years from a cluster of four towns in the Lanusei Valley in the Ogliastra region of the Sardinian province of Nuoro [20]. The study population corresponded to approximately 62% of the population eligible for recruitment in the area. Recruitment of the study population was conducted in 2001. Fasting blood samples of a subgroup of 500 individuals aged ≥65 years were included in our study. All participants signed informed consent to the study protocols approved by the Sardinian Regional Ethics Committee (protocol no. 2171/CE).

### 2.1. Frailty Classification and Multimorbidity

Participants were characterized as robust, pre-frail or frail using the criteria developed by Fried et al. [8]. These include weakness, slow walking speed, low energy expenditure, shrinking and self-reported exhaustion. Participants lacking any of these criteria were classified as robust, participants showing 1–2 criteria were considered pre-frail and those with ≥ 3 criteria were diagnosed as frail. Criteria used for the diagnosis of frailty are listed in detail in Appendix A Table A1. Multimorbidity was defined as the presence of at least two of the following self-reported morbidities: hypertension, diabetes, angina pectoris, stroke, myocardial infarction, cardiac failure and cancer.

### 2.2. Biomarker Analysis

25-Hydroxyvitamin D3 (25-OH-D3): Serum or plasma concentrations of 25-OH-D3 were measured via LC-MS/MS following solid-phase extraction. A detailed description of the method was published previously [21]. All 25-OH-D3 measurements were performed in serum except for the ENRICA cohort, in which only plasma was available. 

Carotenoids, retinol and tocopherols: The carotenoids lutein + zeaxanthin, β-cryptoxanthin, lycopene and α-, β-carotene, as well as the vitamins α-, γ-tocopherol and retinol, were simultaneously analyzed in plasma by using high-performance liquid chromatography (HPLC) with UV and fluorescence detection, as previously described [22]. In brief, plasma (40 µL) was extracted with ethanol/n–butanol (1:1, 200 µL) containing β-apo-carotenal-methyloxime as an internal standard. After centrifugation (21,000× *g*, 15 min at 4 °C), 20 µL of the clear supernatant was analyzed with a Shimadzu Prominence HPLC (LC-20A, Shimadzu, Duisburg, Germany) with chromatographic conditions, as previously described in detail [22].

3-Nitrotyrosine: Protein-bound 3-nitrotyrosine in plasma was measured by using a non-commercial in-house ELISA method, as previously described [23]. 

### 2.3. Statistical Analysis

The participants’ characteristics (Table 1) are described using means and SD for continuous variables (age, body mass index (BMI)) and frequencies (% (*n*)) for categorical variables (sex, smoking status and multimorbidity). To achieve normal distribution, biomarker concentrations needed to be logarithmically transformed. Differences in characteristics and biomarker concentrations between frailty groups were analyzed by using one-way ANOVA (continuous variables) or Pearson’s chi-squared test (distributions). Adjustments of biomarkers’ concentrations for covariates were performed using univariate general linear models (GLM) with frailty status as a fixed factor, with age, sex, body mass index (BMI), multimorbidity and smoking status as covariates and with cohort and season of sampling as random factors (Table 2). Supplemental Figure S1 lists which data needed to be excluded due to missing covariates in the dependency of the statistical analysis performed. Principal component analysis (PCA) based on dietary biomarkers including carotenoids, tocopherols and retinol was conducted to derive biomarker patterns (principal components, PC). The biomarker 25-OH-D3 was not included in the PCA, as there were no intercorrelations between 25-OH-D3 and other biomarkers; this was also due to the small contribution of dietary intake on 25-OH-D3 status. PC were considered for further analyses if they if they possessed an eigenvalue ≥ 1 and explained at least 10% of overall variance (Table 3). Individual factor scores for each PC were calculated and compared between frailty groups by using one-way ANOVA). Finally, multinomial logistic regression analysis was used to investigate cross-sectional associations between biomarker patterns and frailty status. For this purpose, quartiles were formed from the individual factors’ scores of the principal components. The lowest quartile (Q1) was used as the reference category. Adjusted odds ratios (OR) were calculated by adjusting for sociodemographic covariates (age and sex) and health-related covariates (BMI, smoking status, multimorbidity and season of blood collection) as well as for cohort. All statistical analyses were carried out using SPSS software (SPSS Inc., Chicago, IL, USA; Version 25.0.0.2).

## 3. Results

Table 1 displays the participants’ characteristics by frailty status. Complete data on frailty status were available for 1348 participants. In total, 14.8% of the participants were frail and 37.6% were pre-frail; corresponding figures were 11.1% frail and 33.7% pre-frail for men, and 17.0% frail and 40.0% pre-frail for women. Frail participants were significantly older (80.6 ± 6.1 yrs.) than pre-frail (76.2 ± 5.7) and robust participants (73.7 ± 4.6), and they had a higher average BMI than robust participants (29.6 ± 5.6 kg/m² vs. 28.2 ± 4.2). Compared to robust subjects (17.2%), pre-frail (21.6%) and frail (32.0%) subjects were more likely to be multimorbid. The frail and pre-frail subjects’ percentages of active smokers were lower than in robust participants. Detailed participant characteristics according to their cohorts, including missing values for each variable, are displayed in Appendix A Table A2.

Table 2 shows concentrations of biomarkers according to prevalent frailty status. Robust participants had significantly higher plasma concentrations of α-carotene, β-carotene, total carotenoids, lutein + zeaxanthin and β-cryptoxanthin when compared to frail participants (*p* < 0.001), even after adjustment for covariates. Non-adjusted lycopene, retinol, α- and γ-tocopherol showed a U-shaped tendency, being higher in robust and frail participants than in pre-frail subjects, whereas after adjustment, values decreased from robustness to frailty, except for γ-tocopherol. No associations between vitamin E isoforms, vitamin A, vitamin D3 or oxidative stress and frailty status were found after adjusting for covariates.

The PCA revealed two dominant dietary biomarker patterns within the study population (Table 3). The first one (PC1) explained 36.4% of total variance and was characterized by high factor loadings for all carotenoids, tocopherols and retinol. 

PC1 might be explained by a diet high in fruits and vegetables. The PC2 explained 20.9% of total variance and showed high positive factor loadings for tocopherols and retinol as well as low positive factor loadings for lycopene while simultaneously showing negative factor loadings for α-carotene, β-carotene, lutein + zeaxanthin and β-cryptoxanthin. Here, positive factor loadings might mainly result from a high consumption of animal and tomato products, whereas negative factor loadings possibly reflect a low consumption of fruits and vegetables.

**Figure 1:**
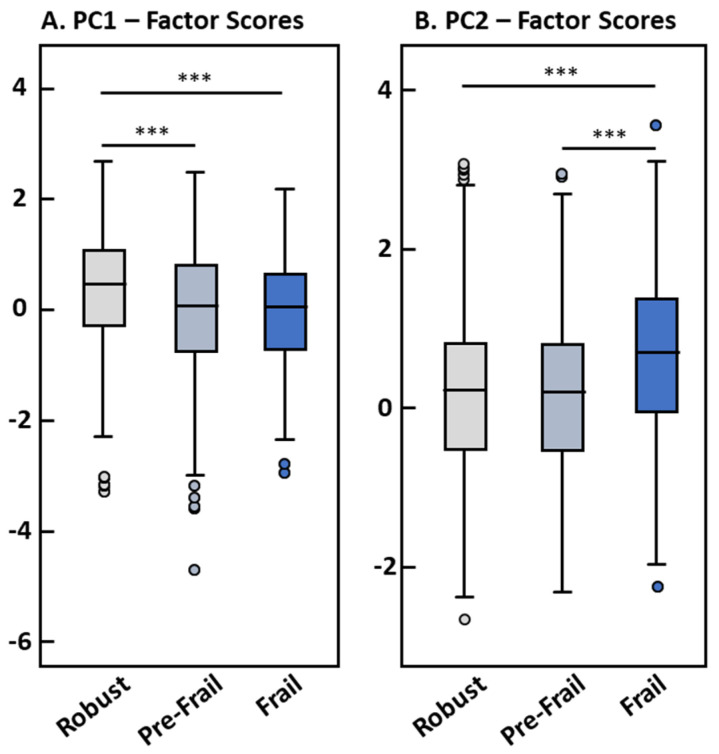
Factor scores of PC1 (**A**) and PC2 (**B**) by frailty status (*n* = 1348). Differences between frailty groups were assessed using one-way ANOVA. *** significance level *p* < 0.001.

Robust participants had significantly higher positive factor scores for PC1 than pre-frail and frail subjects (means: 0.22 ± 1.01 vs. −0.12 ± 1.11 and −0.11 ± 0.93, respectively; both comparisons *p* < 0.001), whereas no difference between frail and pre-frail participants was observed (Figure 1A). At the same time, frail participants showed significantly higher factor scores for PC2 than pre-frail and robust subjects (0.56 ± 1.03 vs. 0.04 ± 0.99 and 0.06 ± 1.00, respectively; both comparisons *p* < 0.001) with no differences between robust and pre-frail participants (Figure 1B). 

**Figure 2:**
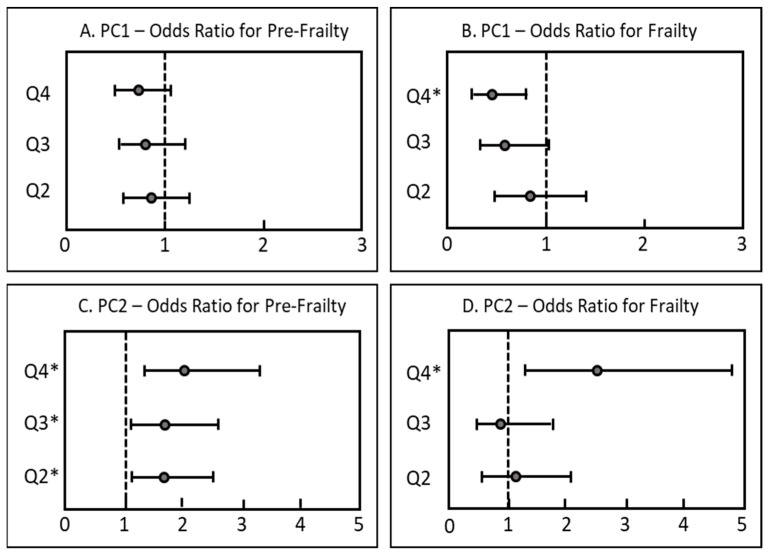
Cross-sectional associations between patterns of fat-soluble biomarkers (considered as quartiles) and frailty status in older adults (*n* =1271). Results are displayed as odds ratios (OR with 95% CI). ORs were determined via multinomial regression analysis adjusted for season of blood sampling, sex, age, BMI, current smoking status, multimorbidity and cohort; the vertical line at OR = 1 represents the reference odds ratio to be considered robust; * *p* < 0.05. Q1 = reference quartile. (**A**): Odds ratios for pre-frailty by PC1 quartile. (**B**): Odds ratios for frailty by PC1 quartile. (**C**): Odds ratios for pre-frailty by PC2 quartile. (**D**): Odds ratios for frailty by PC2 quartile.

Participants in the highest PC1 quartile had significantly lower odds (OR 0.45, 95% CI: 0.25–0.80, *p*-value 0.006) of being frail compared to the lowest quartile (Figure 2). For PC2, participants in the highest quartile had significantly higher odds (OR 2.48, 95% CI: 1.28–4.80, *p*-value 0.007) of being frail than the lowest quartile. Interestingly, participants in the second, third and fourth quartile of PC2 had higher odds for pre-frailty compared to the lowest quartile, whereas no such associations between PC1 quartiles and pre-frailty participants were observed.

## 4. Discussion

In our study, we aimed to confirm cross-sectional associations between fat-soluble micronutrients and frailty statuses previously found in four independent European cohorts of people aged 65+ years [14]. Compared to the cohorts of the first phase of the FRAILOMIC project, the prevalence of pre-frailty (37.6% vs. 41.7%) was similar, whereas the prevalence of frailty (14.8% vs. 22.1%) was lower in the current study [14]. This can be explained by the low prevalence of frailty in the EXERNET (4.9%) cohort. The prevalence of frailty and pre-frailty in the current study are consistent with those reported in previous studies, ranging from 34.0% to 52.8% for pre-frailty and from 3.0% to 15.6% for frailty among 60,816 individuals aged ≥50 years from 18 European countries [24]. Likewise, as reported in the first phase of FRAILOMIC and in other studies, frailty was associated with age, female sex, BMI and multimorbidity [14,24,25,26]. Age, sex, BMI, multimorbidity and smoking status were therefore considered as frailty-associated covariates and were adjusted for in our statistical analyses. 

We observed inverse associations of plasma levels of the carotenoids β-carotene, β-cryptoxanthin and lutein + zeaxanthin with frailty status as also found in the first project phase [14]. Although frail subjects from the first phase had significantly lower γ-tocopherol levels, no associations occurred between vitamin E isoforms and frailty status in the present study. Using a machine learning approach with data from the first FRAILOMIC phase, lutein + zeaxanthin, vitamin D3 and troponin T were identified as potential biomarkers for frailty, whereas no association between retinol and frailty was found [15]. We revealed that a biomarker pattern characterized by high levels of carotenoids and tocopherols (PC1) was associated with a lower frequency of prevalent frailty. The reason for this might be due to the antioxidant and anti-inflammatory properties of carotenoids [27]. Carotenoids are known to be effective scavengers of reactive oxygen species, being one of the main causes for oxidative stress. Increased oxidative stress in combination with subsequent inflammation have repeatedly been associated with frailty status [28,29,30]. A higher dietary intake of antioxidative phytochemicals, such as carotenoids, might counteract the oxidative imbalance in muscle tissues that occurs after exercise [31]. Thus, higher dietary intake and plasma levels of carotenoids are associated with improved grip strength and walking speed [32,33]. A pro-inflammatory diet, as assessed with the Dietary Inflammatory Index DII, was associated with higher systemic inflammation and slower gait speed as well as lower muscle mass in old adults [34]. A dietary biomarker pattern composed of low levels of carotenoids and high levels of vitamin E, A and lycopene (PC2) was positively associated with frailty status in the current study. This biomarker pattern can be mainly explained by a diet rich in animal-based foods, those being the main source for vitamin A and E, whereas the high plasma lycopene levels with low overall carotenoid status can be explained by high consumption of processed and unprocessed tomatoes, which are products that are good sources of lycopene but are low in other carotenoids. Adherence to a Westernized diet rich in processed meats, saturated fats, refined grains, sugar, alcohol and salt and low in fruits and vegetable intake was associated with increased frailty risk, whereas a Mediterranean-style diet has been repeatedly associated with a reduced risk of frailty [35,36,37]. A high consumption level of red meat, especially processed, was associated with a higher risk of frailty in older women in the Nurses’ Health Study [38]. Additionally, the risk for frailty was reduced when one serving/day of unprocessed or processed red meat was replaced with alternative protein sources such as nuts, fish or legumes [38]. Furthermore, higher intakes of plant protein, but not animal protein or diary protein, was associated with lower risk of frailty in the Nurses’ Health Study [39]. However, the positive associations of fruit and vegetable consumption with frailty could be attributed to other components found in those foods such as fiber and potassium. Whereas a diet rich in fiber is linked to a lower risk of cardiovascular diseases and obesity, both being risk factors for frailty, a diet high in potassium contributes to the maintenance of normal muscle function [40,41]. In addition to possible dietary factors, low levels of fat-soluble micronutrients can also be a consequence of acute or chronic inflammation. Thus, Kochlik et al. recently showed that frail patients could be characterized by a blood pattern that simultaneously shows low concentrations of fat-soluble micronutrients and elevated concentrations of pro-inflammatory cytokines [12].

Interestingly, Pilleron et al. observed contrary results to those in the first phase of the FRAILOMIC initiative, reporting that lower levels of vitamin E and A and high levels of carotenoids increased odds for frailty [42]. However, the PCA conducted by Pilleron et al., in addition to carotenoids and tocopherols, also included 25-OH-D3 [42], which was not regarded as a dietary biomarker in the current analysis, as only about 10% of vitamin D3 is derived from dietary intake [43]. Furthermore, our PCA revealed no intercorrelations between 25-OH-D3 and other measured fat soluble-micronutrients. Thus, PCAs are not comparable between these two studies because they were based on different biomarkers. 

In our study, no association between 25-OH-D3 and frailty was shown (*p* = 0.125) other than as described in the first FRAILOMIC phase and in other studies [14,44]. This is likely due to a proportionally higher number of supplement users in the frail group compared to the robust group. Thus, the relative number of individuals with vitamin D3 levels > 150 nM was higher in the frail and pre-frail groups than in the robust group, even when blood sampling was performed in the winter months (December to March). 

Associations between dietary biomarker patterns and pre-frailty showed varying results. Whereas no association between PC1 quartiles and pre-frailty was observed, participants in the highest three quartiles of PC2 had significantly increased frequencies of pre-frailty (Figure 2). Therefore, no significant differences in frailty between the highest three quartiles were shown. This might be due to a more heterogenic nature of the pre-frailty group. As only one frailty criterion is sufficient to be characterized as pre-frail, the probability of false positive diagnoses in this group is increased. As frailty classification using the frailty phenotype is mostly based on self-reported criteria, a biomarker-based frailty diagnosis might thereby be a more helpful tool for an objective diagnosis of frailty despite its higher cost.

The present study has several limitations. Five-year follow-up data on frailty status were lacking among most cohorts, which did not allow assessment of the role of dietary biomarker patterns on incident frailty. Additional covariates such as medication intake were only available in the TSHA and ENRICA studies’ data and were therefore not included in the cross-sectional analysis. A higher medication intake was shown to be negatively associated with plasma carotenoids and positively with vitamin E and A [45]. Information on frailty status for the SardiNIA cohort could not be used, as frailty status was assessed differently than in the other cohorts. Unfortunately, data on supplement use were not available for each cohort, and therefore, analyses could not be adjusted for supplement intake. In addition, dietary intake was not available except in the ENRICA study; thus, the calculated biomarker patterns could not be statically adjusted for diet-related factors (e.g., energy intake). The validation of the phase 1 research results in four independent cohorts is the strength of our study, resulting in consistent findings based on data from a total of eight studies from three European countries with over 2700 participants aged 65+ years. As our study subjects originated from different European countries, our results cover a wide range of dietary and lifestyle habits. Biomarker analyses were furthermore performed in one laboratory in a blinded fashion, limiting variability related to operators, methods and instruments.

## 5. Conclusions

In this validation study, we confirmed previously found inverse associations between carotenoids, especially β-carotene, lutein + zeaxanthin and β-cryptoxanthin, and frailty status in four independent European cohorts. In contrast, biomarker patterns higher in vitamin E, A and lycopene and lower in carotenoids were positively associated with frailty. We conclude that an adequate consumption of carotenoid-rich diets might diminish frailty risk, whereas diets low in α-carotene, β-carotene, lutein + zeaxanthin, and β-cryptoxanthin might promote frailty risk. Considering dietary markers (e.g., fruit and vegetable consumption) in novel frailty indexes might be of future interest for a comprehensive diagnosis of physical frailty. 

## Figures and Tables

**Table 1:** Participant characteristics.

	Total	Robust	Pre-Frail	Frail	*p*-Value
N (%)	1348	642 (47.6)	507 (37.6)	199 (14.8)	-
Females, % (*n*)	62.0 (852)	55.8 (358)	65.9 (334)	71.4 (142)	<0.001
Age, years	75.64 ± 5.95	73.65 ± 5.01 ^a^	76.24 ± 5.73 ^b^	80.55 ± 6.13 ^c^	<0.001
BMI, kg/m^2^	28.82 ± 4.61	28.21 ± 4.23 ^a^	29.28 ± 4.55 ^b^	29.55 ± 5.59 ^b^	<0.001
Current smoker, % (*n*)	7.0 (93)	8.9 (56)	5.4 (27)	5.0 (10)	0.039
Multimorbidity, % (*n*)	21.1 (275)	17.2 (107)	21.6 (106)	32.0 (62)	<0.001

Results reported as means ± standard deviation or % (*n*). Values sharing a common superscript letter are not significantly different (difference between frailty groups found by using one-way ANOVA). Differences between distributions were assessed using Pearson’s χ^2^ test. BMI: body mass index

**Table 2:** Biomarker concentrations in dependency of frailty status.

Biomarker	Total	Robust	Pre-Frail	Frail	*p*-Value
Total Carotenoids (µM)	2.24 (2.16–2.32)	2.58 (2.46–2.71) ^a^	2.00 (1.88–2.21) ^b^	1.91 (1.75–2.08) ^c^	<0.001
adjusted		2.57 (2.44–2.70) ^a^	2.17 (2.04–2.30) ^b^	1.96 (1.75–2.20) ^b^	<0.001
α-Carotene (µM)	0.09 (0.09–0.10)	0.10 (0.10–0.11) ^a^	0.09 (0.08–0.09) ^b^	0.09 (0.07–0.09) ^b^	0.001
adjusted		0.10 (0.09–0.11)	0.09 (0.08–0.10)	0.09 (0.08–0.10)	0.042
β-Carotene (µM)	0.29 (0.27–0.30)	0.33 (0.31–0.36) ^a^	0.26 (0.24–0.28) ^b^	0.23 (0.19–26) ^b^	<0.001
adjusted		0.32 (0.30–0.35) ^a^	0.27 (0.25–0.29 ^b^	0.24 (0.20–0.28) ^b^	0.001
Lycopene (µM)	0.99 (0.94–1.05)	1.18 (1.10–1.27) ^a^	0.82 (0.75–0.91) ^b^	0.89 (0.78–1.00) ^b^	<0.001
adjusted		1.17 (1.08–1.25) ^a^	0.99 (0.90–1.08) ^b^	0.94 (0.79–1.11) ^a^	0.006
Lutein + Zeaxanthin (µM)	0.22 (0.21–0.23)	0.24 (0.23–0.26) ^a^	0.21 (0.20–0.22) ^b^	0.18 (0.16–0.20) ^b^	<0.001
adjusted		0.24 (0.23–0.25) ^a^	0.21 (0.20–0.23) ^a,b^	0.18 (0.16–0.20) ^b^	<0.001
β-Cryptoxanthin (µM)	0.33 (0.32–0.35)	0.37 (0.35–0.40) ^a^	0.33 (0.31–0.36) ^a^	0.23 (0.20–0.27) ^b^	<0.001
adjusted		0.39 (0.36–0.42) ^a^	0.30 (0.27–0.33) ^b^	0.25 (0.21–0.30) ^b^	<0.001
Retinol (µM)	1.69 (1.65–1.73)	1.71 (1.66–1.77)	1.63 (1.57–1.69)	1.69 (1.65–1.73)	0.007
adjusted		1.75 (1.70–1.80)	1.73 (1.68–1.79)	1.66 (1.56–1.77)	0.407
α-Tocopherol (µM)	38.0 (37.3–38.7)	38.6 (37.7–39.6) ^a^	36.15 (35.0–37.2) ^b^	40.9 (39.4–42.5) ^a^	<0.001
adjusted		39.2 (38.4–40.0)	37.9 (37.1–38.8)	37.9 (36.2–39.6)	0.097
γ-Tocopherol (µM)	0.92 (0.89–0.95)	0.95 (0.91–1.00) ^a^	0.84 (0.80–0.89) ^b^	1.01 (0.95–1.08) ^a^	<0.001
adjusted		0.95 (0.91–0.99)	0.93 (0.88–0.97)	0.99 (0.90–1.09)	0.475
25-OH-D3 (nM)	54.0 (52.5–55.6)	57.7 (55.5–60.0) ^a^	52.9 (50.5–55.4)^b^	45.9 (42.0–50.2) ^c^	<0.001
adjusted		55.0 (52.5–57.7)	51.8 (49.1–54.7)	49.8 (44.9–55.3)	0.125
3-Nitrotyrosine (pmol/mg)	3.62 (3.45–3.79)	3.49 (3.27–3.72)	3.59 (3.31–3.88)	4.19 (3.68–4.77)	0.008
adjusted		3.61 (3.37–3.86)	3.49 (3.22–3.78)	3.88 (3.33–4.52)	0.466

Results reported as geometric means (95% confidence interval; CI). Values sharing a common superscript letter are not significantly different (differences between frailty groups by GLM). Unadjusted model (*n* = 1271); adjusted model (*n* = 1271). Adjustments included season of blood sampling, sex, age, BMI, current smoking status, multimorbidity and cohort.

**Table 3:** Factor loadings for principal components 1 and 2 (*n*= 1884).

PC1 Biomarkers	PC1 Factor Loadings	PC2 Biomarkers	PC2 Factor Loadings
β-Carotene	0.732	Retinol	0.601
α-Tocopherol	0.693	γ-Tocopherol	0.572
α-Carotene	0.648	α-Tocopherol	0.506
Lutein + zeaxanthin	0.598	Lycopene	0.161
Lycopene	0.583	α-Carotene	*−0.473*
β-Cryptoxanthin	0.523	β-Carotene	*−0.458*
γ-Tocopherol	0.507	β-Cryptoxanthin	*−0.437*
Retinol	0.500	Lutein + zeaxanthin	*−0.273*
Variance explained [%]	36.4	Variance explained [%]	20.9

Factor loadings written in italics represent negative loadings.

## Data Availability

The data that support the findings of this study are available from the corresponding author and the scientists responsible for the respective cohorts, upon reasonable request.

## Supplementary Materials

The following supporting information can be downloaded at: https://www.mdpi.com/article/10.3390/nu15051142/s1, Figure S1: Flow chart showing the exclusion criteria in dependency of the statistical analysis performed.

## Appendix A

**Table A1:** Harmonized frailty assessment used in the FRAILOMIC Initiative.

Criteria	Characteristic
Slowness	Defined as the worst quintile in the three-meter walking speed test, adjusted for sex and height
Inactivity	Defined as the worst quintile in the PASE score
Shrinking, weight loss	Unintentional weight loss of ≥4.5 kg within a year
Weakness	Defined as the worst quintile of maximum grip strength on the dominant hand, adjusted for sex and body mass index
Exhaustion	Self-reported exhaustion (CES-D depression scale)

CES-D: Center for Epidemiologic Studies Depression Scale; PASE: Physical Activity Scale for the Elderly.

**Table A2:** Detailed participants’ characteristics by cohort.

	Total	ENRICA	TSHA	EXERNET	SardiNIA	*p*-Value
**Country**		Spain	Spain	Spain	Italy	
N (%)	1927	498 (30.5)	498 (30.5)	431 (26.4)	500 (12.6)	
**Age (years)**	75.32 ± 5.57	74.66 ± 6.08	75.86 ± 6.64	76.86 ± 4.54	74.29 ± 4.01	<0.001
Missing, *n*	52	1	0	51	0	
**Sex, *n* (%)**						<0.001
Female	1124 (59.9)	285 (57.3)	281 (56.4)	286 (75.3)	272 (54.4)	
Male	751 (40.1)	212 (42.7)	217 (43.6)	94 (24.7)	228 (45.6)	
Missing, *n*	52	1	0	51	0	
**BMI, kg/m^2^**	28.60 ± 4.51	28.23 ± 4.56	29.35 ± 4.98	28.86 ± 3.86	28.04 ± 4.20	<0.001
<25 kg/m^2^, *n* (%)	369 (19.1)	119 (24.0)	88 (17.7)	57 (15.4)	105 (21.4)	
25–29.9 kg/m^2^, *n* (%)	861 (46.5)	221 (44.6)	210 (42.3)	183 (49.6)	247 (50.3)	
≥30 kg/m^2^, *n* (%)	622 (33.6)	155 (31.3)	199 (40.0)	129 (35.0)	139 (28.3)	
Missing, *n*	75	3	1	62	9	
**Frailty status, *n* (%)**						<0.001
Robust	642 (47.6)	203 (41.8)	270 (54.3)	169 (46.3)	-	
Pre-frail	507 (37.6)	195 (40.1)	134 (27.0)	178 (48.8)	-	
Frail	199 (14.8)	88 (18.1)	93 (18.7)	18 (4.9)	-	
Missing, *n*	579	12	1	66	500	
**Smoking status, *n* (%)**						<0.001
Current	115 (6.2)	44 (8.8)	40 (8.0)	10 (2.8)	21 (4.3)	
Past	521 (28.2)	186 (37.5)	133 (26.7)	60 (16.8)	142 (28.7)	
Never	1210 (65.5)	266 (53.6)	325 (65.3)	288 (80.4)	331 (67.0)	
Missing, *n*	81	2	0	73	6	
**Health status, *n* (%)**						
Multimorbidity	396 (21.9)	99 (20.2)	126 (25.7)	58 (16.3)	113 (23.5)	<0.001
Missing, *n*	115	8	8	80	19	
**Biomarkers**						
25-OH-D3 (nM)	56.51 ± 33.74	58.36 ± 32.16	62.97 ± 34.75	64.85 ± 37.00	41.23 ± 25.50	<0.001
25-OH-D3: <25 nM, *n* (%)	142 (8.8)	40 (8.0)	42 (8.5)	36 (8.5)	24 (11.9)	
25-OH-D3: 25–49.9 nM, *n* (%)	535 (33.1)	188 (37.8)	149 (30.1)	128 (30.3)	70 (34.7)	
25-OH-D3: ≥50 nM, *n* (%)	939 (57.5)	269 (54.1)	304 (61.4)	258 (61.1)	108 (53.5)	
Total Carotenoids (µM)	2.96 ± 1.70	3.09 ± 1.63	3.09 ± 1.70	1.70 ± 0.93	3.77 ± 1.67	<0.001
α-Carotene (µM)	0.12 ± 0.13	0.17 ± 0.14	0.12 ± 0.13	0.12 ± 0.12	0.09 ± 0.09	<0.001
β-Carotene (µM)	0.41 ± 0.36	0.45 ± 0.37	0.43 ± 0.36	0.31 ± 0.28	0.45 ± 0.40	< 0.001
Lycopene (µM)	1.64 ± 1.23	1.56 ± 1.09	1.91 ± 1.23	0.57 ± 0.41	2.39 ± 1.18	< 0.001
Lutein + Zeaxanthin (µM)	0.29 ± 0.19	0.32 ± 0.22	0.24 ± 0.13	0.24 ± 0.17	0.35 ± 0.19	< 0.001
β-Cryptoxanthin (µM)	0.49 ± 0.47	0.59 ± 0.54	0.40 ± 0.37	0.46 ± 0.36	0.50 ± 0.55	< 0.001
Retinol (µM)	1.78 ± 0.65	2.04 ± 0.57	2.10 ± 0.67	1.17 ± 0.38	1.71 ± 0.48	< 0.001
α-Tocopherol (µM)	36.81 ± 12.02	46.27 ± 9.72	43.61 ± 10.08	27.01 ± 6.42	28.97 ± 6.88	< 0.001
γ-Tocopherol (µM)	1.00 ± 0.63	1.22 ± 0.64	1.24 ± 0.68	0.66 ± 0.37	0.81 ± 0.53	< 0.001
3-Nitrotyrosine (pmol/mg)	4.76 ± 3.67	7.01 ± 4.00	3.54 ± 2.49	3.67 ± 3.24	-	< 0.001

Values are displayed as means ± SD unless otherwise stated. Differences between frailty groups were assessed using one-way ANOVA for continuous variables and using Pearson’s χ^2^ test for distributions. *p* < 0.05. Multimorbidity: see Section 2.1. for definition.

## References

[1] Population Division
of the United Nations Department of Economic and Social Affairs World Population Ageing 2020 Highlights: Living Arrangements of Older Persons (ST/ESA/SER.A/451. World Population Ageing 2020 Highlights. https://www.un.org/development/desa/pd/sites/www.un.org.development.desa.pd/files/files/documents/2020/Sep/un_pop_2020_pf_ageing_10_key_messages.pdf.

[2] Kojima G. (2015). Frailty as a Predictor of Future Falls Among Community-Dwelling Older People: A Systematic Review and Meta-Analysis. J. Am. Med. Dir. Assoc..

[3] Kojima G. (2016). Frailty as a predictor of fractures among community-dwelling older people: A systematic review and meta-analysis. Bone.

[4] Yang X., Lupon J., Vidan M.T., Ferguson C., Gastelurrutia P., Newton P.J., Macdonald P.S., Bueno H., Bayes-Genis A., Woo J. (2018). Impact of Frailty on Mortality and Hospitalization in Chronic Heart Failure: A Systematic Review and Meta-Analysis. J. Am. Heart Assoc..

[5] Vermeiren S., Vella-Azzopardi R., Beckwee D., Habbig A.K., Scafoglieri A., Jansen B., Bautmans I., Gerontopole Brussels Study g. (2016). Frailty and the Prediction of Negative Health Outcomes: A Meta-Analysis. J. Am. Med. Dir. Assoc..

[6] Rodriguez-Manas L., Feart C., Mann G., Vina J., Chatterji S., Chodzko-Zajko W., Gonzalez-Colaco Harmand M., Bergman H., Carcaillon L., Nicholson C. (2013). Searching for an operational definition of frailty: A Delphi method based consensus statement: The frailty operative definition-consensus conference project. J. Gerontol. A Biol. Sci. Med. Sci..

[7] Gordon A.L., Masud T., Gladman J.R. (2014). Now that we have a definition for physical frailty, what shape should frailty medicine take?. Age Ageing.

[8] Fried L.P., Tangen C.M., Walston J., Newman A.B., Hirsch C., Gottdiener J., Seeman T., Tracy R., Kop W.J., Burke G. (2001). Frailty in older adults: Evidence for a phenotype. J. Gerontol. A Biol. Sci. Med. Sci..

[9] Searle S.D., Mitnitski A., Gahbauer E.A., Gill T.M., Rockwood K. (2008). A standard procedure for creating a frailty index. BMC Geriatr..

[10] Mitnitski A., Collerton J., Martin-Ruiz C., Jagger C., von Zglinicki T., Rockwood K., Kirkwood T.B. (2015). Age-related frailty and its association with biological markers of ageing. BMC Med..

[11] Sepulveda M., Arauna D., Garcia F., Albala C., Palomo I., Fuentes E. (2022). Frailty in Aging and the Search for the Optimal Biomarker: A Review. Biomedicines.

[12] Kochlik B., Franz K., Henning T., Weber D., Wernitz A., Herpich C., Jannasch F., Aykac V., Muller-Werdan U., Schulze M.B. (2022). Frailty is characterized by biomarker patterns reflecting inflammation or muscle catabolism in multi-morbid patients. J. Cachexia Sarcopenia Muscle.

[13] Erusalimsky J.D., Grillari J., Grune T., Jansen-Duerr P., Lippi G., Sinclair A.J., Tegner J., Vina J., Durrance-Bagale A., Minambres R. (2016). In Search of ‘Omics’-Based Biomarkers to Predict Risk of Frailty and Its Consequences in Older Individuals: The FRAILOMIC Initiative. Gerontology.

[14] Kochlik B., Stuetz W., Peres K., Pilleron S., Feart C., Garcia Garcia F.J., Bandinelli S., Gomez-Cabrero D., Rodriguez-Manas L., Grune T. (2019). Associations of fat-soluble micronutrients and redox biomarkers with frailty status in the FRAILOMIC initiative. J. Cachexia Sarcopenia Muscle.

[15] Gomez-Cabrero D., Walter S., Abugessaisa I., Minambres-Herraiz R., Palomares L.B., Butcher L., Erusalimsky J.D., Garcia-Garcia F.J., Carnicero J., Hardman T.C. (2021). A robust machine learning framework to identify signatures for frailty: A nested case-control study in four aging European cohorts. Geroscience.

[16] Garcia-Garcia F.J., Gutierrez Avila G., Alfaro-Acha A., Amor Andres M.S., De Los Angeles De La Torre Lanza M., Escribano Aparicio M.V., Humanes Aparicio S., Larrion Zugasti J.L., Gomez-Serranillo Reus M., Rodriguez-Artalejo F. (2011). The prevalence of frailty syndrome in an older population from Spain. The Toledo Study for Healthy Aging. J. Nutr. Health Aging.

[17] Rodriguez-Artalejo F., Graciani A., Guallar-Castillon P., Leon-Munoz L.M., Zuluaga M.C., Lopez-Garcia E., Gutierrez-Fisac J.L., Taboada J.M., Aguilera M.T., Regidor E. (2011). Rationale and methods of the study on nutrition and cardiovascular risk in Spain (ENRICA). Rev. Esp. Cardiol..

[18] Gomez-Cabello A., Vicente-Rodriguez G., Albers U., Mata E., Rodriguez-Marroyo J.A., Olivares P.R., Gusi N., Villa G., Aznar S., Gonzalez-Gross M. (2012). Harmonization process and reliability assessment of anthropometric measurements in the elderly EXERNET multi-centre study. PLoS ONE.

[19] Pedrero-Chamizo R., Gomez-Cabello A., Delgado S., Rodriguez-Llarena S., Rodriguez-Marroyo J.A., Cabanillas E., Melendez A., Vicente-Rodriguez G., Aznar S., Villa G. (2012). Physical fitness levels among independent non-institutionalized Spanish elderly: The elderly EXERNET multi-center study. Arch. Gerontol. Geriatr..

[20] Pilia G., Chen W.M., Scuteri A., Orru M., Albai G., Dei M., Lai S., Usala G., Lai M., Loi P. (2006). Heritability of cardiovascular and personality traits in 6,148 Sardinians. PLoS Genet..

[21] Henning T., Kochlik B., Kusch P., Strauss M., Juric V., Pignitter M., Marusch F., Grune T., Weber D. (2022). Pre-Operative Assessment of Micronutrients, Amino Acids, Phospholipids and Oxidative Stress in Bariatric Surgery Candidates. Antioxidants.

[22] Stuetz W., Weber D., Dolle M.E., Jansen E., Grubeck-Loebenstein B., Fiegl S., Toussaint O., Bernhardt J., Gonos E.S., Franceschi C. (2016). Plasma Carotenoids, Tocopherols, and Retinol in the Age-Stratified (35-74 Years) General Population: A Cross-Sectional Study in Six European Countries. Nutrients.

[23] Weber D., Stuetz W., Bernhard W., Franz A., Raith M., Grune T., Breusing N. (2014). Oxidative stress markers and micronutrients in maternal and cord blood in relation to neonatal outcome. Eur. J. Clin. Nutr..

[24] Manfredi G., Midao L., Paul C., Cena C., Duarte M., Costa E. (2019). Prevalence of frailty status among the European elderly population: Findings from the Survey of Health, Aging and Retirement in Europe. Geriatr. Gerontol. Int..

[25] Gagesch M., Chocano-Bedoya P.O., Abderhalden L.A., Freystaetter G., Sadlon A., Kanis J.A., Kressig R.W., Guyonnet S., DaSilva J.A.P., Felsenberg D. (2022). Prevalence of Physical Frailty: Results from the DO-HEALTH Study. J. Frailty Aging.

[26] Hanlon P., Nicholl B.I., Jani B.D., Lee D., McQueenie R., Mair F.S. (2018). Frailty and pre-frailty in middle-aged and older adults and its association with multimorbidity and mortality: A prospective analysis of 493 737 UK Biobank participants. Lancet Public Health.

[27] Saini R.K., Nile S.H., Park S.W. (2015). Carotenoids from fruits and vegetables: Chemistry, analysis, occurrence, bioavailability and biological activities. Food Res. Int..

[28] Bernabeu-Wittel M., Gomez-Diaz R., Gonzalez-Molina A., Vidal-Serrano S., Diez-Manglano J., Salgado F., Soto-Martin M., Ollero-Baturone M., on behalf of the Proteo Researchers (2020). Oxidative Stress, Telomere Shortening, and Apoptosis Associated to Sarcopenia and Frailty in Patients with Multimorbidity. J. Clin. Med..

[29] Mu L., Jiang L., Chen J., Xiao M., Wang W., Liu P., Wu J. (2021). Serum Inflammatory Factors and Oxidative Stress Factors Are Associated with Increased Risk of Frailty and Cognitive Frailty in Patients with Cerebral Small Vessel Disease. Front. Neurol..

[30] El Assar M., Angulo J., Rodriguez-Manas L. (2020). Frailty as a phenotypic manifestation of underlying oxidative stress. Free Radic. Biol. Med..

[31] Powers S.K., Jackson M.J. (2008). Exercise-induced oxidative stress: Cellular mechanisms and impact on muscle force production. Physiol. Rev..

[32] Sahni S., Dufour A.B., Fielding R.A., Newman A.B., Kiel D.P., Hannan M.T., Jacques P.F. (2021). Total carotenoid intake is associated with reduced loss of grip strength and gait speed over time in adults: The Framingham Offspring Study. Am. J. Clin. Nutr..

[33] Semba R.D., Blaum C., Guralnik J.M., Moncrief D.T., Ricks M.O., Fried L.P. (2003). Carotenoid and vitamin E status are associated with indicators of sarcopenia among older women living in the community. Aging Clin. Exp. Res..

[34] Hass U., Herpich C., Kochlik B., Weber D., Grune T., Norman K. (2022). Dietary Inflammatory Index and Cross-Sectional Associations with Inflammation, Muscle Mass and Function in Healthy Old Adults. J. Nutr. Health Aging.

[35] Leon-Munoz L.M., Garcia-Esquinas E., Lopez-Garcia E., Banegas J.R., Rodriguez-Artalejo F. (2015). Major dietary patterns and risk of frailty in older adults: A prospective cohort study. BMC Med..

[36] Talegawkar S.A., Bandinelli S., Bandeen-Roche K., Chen P., Milaneschi Y., Tanaka T., Semba R.D., Guralnik J.M., Ferrucci L. (2012). A higher adherence to a Mediterranean-style diet is inversely associated with the development of frailty in community-dwelling elderly men and women. J. Nutr..

[37] Bollwein J., Diekmann R., Kaiser M.J., Bauer J.M., Uter W., Sieber C.C., Volkert D. (2013). Dietary quality is related to frailty in community-dwelling older adults. J. Gerontol. A Biol. Sci. Med. Sci..

[38] Struijk E.A., Fung T.T., Sotos-Prieto M., Rodriguez-Artalejo F., Willett W.C., Hu F.B., Lopez-Garcia E. (2022). Red meat consumption and risk of frailty in older women. J. Cachexia Sarcopenia Muscle.

[39] Struijk E.A., Fung T.T., Rodriguez-Artalejo F., Bischoff-Ferrari H.A., Hu F.B., Willett W.C., Lopez-Garcia E. (2022). Protein intake and risk of frailty among older women in the Nurses’ Health Study. J. Cachexia Sarcopenia Muscle.

[40] Slavin J.L., Lloyd B. (2012). Health benefits of fruits and vegetables. Adv. Nutr..

[41] Dawson-Hughes B., Harris S.S., Ceglia L. (2008). Alkaline diets favor lean tissue mass in older adults. Am. J. Clin. Nutr..

[42] Pilleron S., Weber D., Peres K., Colpo M., Gomez-Cabrero D., Stuetz W., Dartigues J.F., Ferrucci L., Bandinelli S., Garcia-Garcia F.J. (2019). Patterns of circulating fat-soluble vitamins and carotenoids and risk of frailty in four European cohorts of older adults. Eur. J. Nutr..

[43] Martens M.C., Emmert S., Boeckmann L. (2020). Sunlight, Vitamin D, and Xeroderma Pigmentosum. Adv. Exp. Med. Biol..

[44] Marcos-Perez D., Sanchez-Flores M., Proietti S., Bonassi S., Costa S., Teixeira J.P., Fernandez-Tajes J., Pasaro E., Valdiglesias V., Laffon B. (2020). Low Vitamin D Levels and Frailty Status in Older Adults: A Systematic Review and Meta-Analysis. Nutrients.

[45] Weber D., Kochlik B., Stuetz W., Dolle M.E.T., Jansen E., Grubeck-Loebenstein B., Debacq-Chainiaux F., Bernhardt J., Gonos E.S., Capri M. (2020). Medication Intake Is Associated with Lower Plasma Carotenoids and Higher Fat-Soluble Vitamins in the Cross-Sectional MARK-AGE Study in Older Individuals. J. Clin. Med..

