# Supplementary material for: Patterns of Dietary Blood Markers Are Related to Frailty Status in the FRAILOMIC Validation Phase
Source: Nutrients. 2023 Feb 24;15(5):1142. doi: 10.3390/nu15051142 (PMC10005398; doi:10.3390/nu15051142)
Supplement: Supplementary file 1 [file nutrients-15-01142-s001.zip › nutrients-2183891-supplementary.pdf]

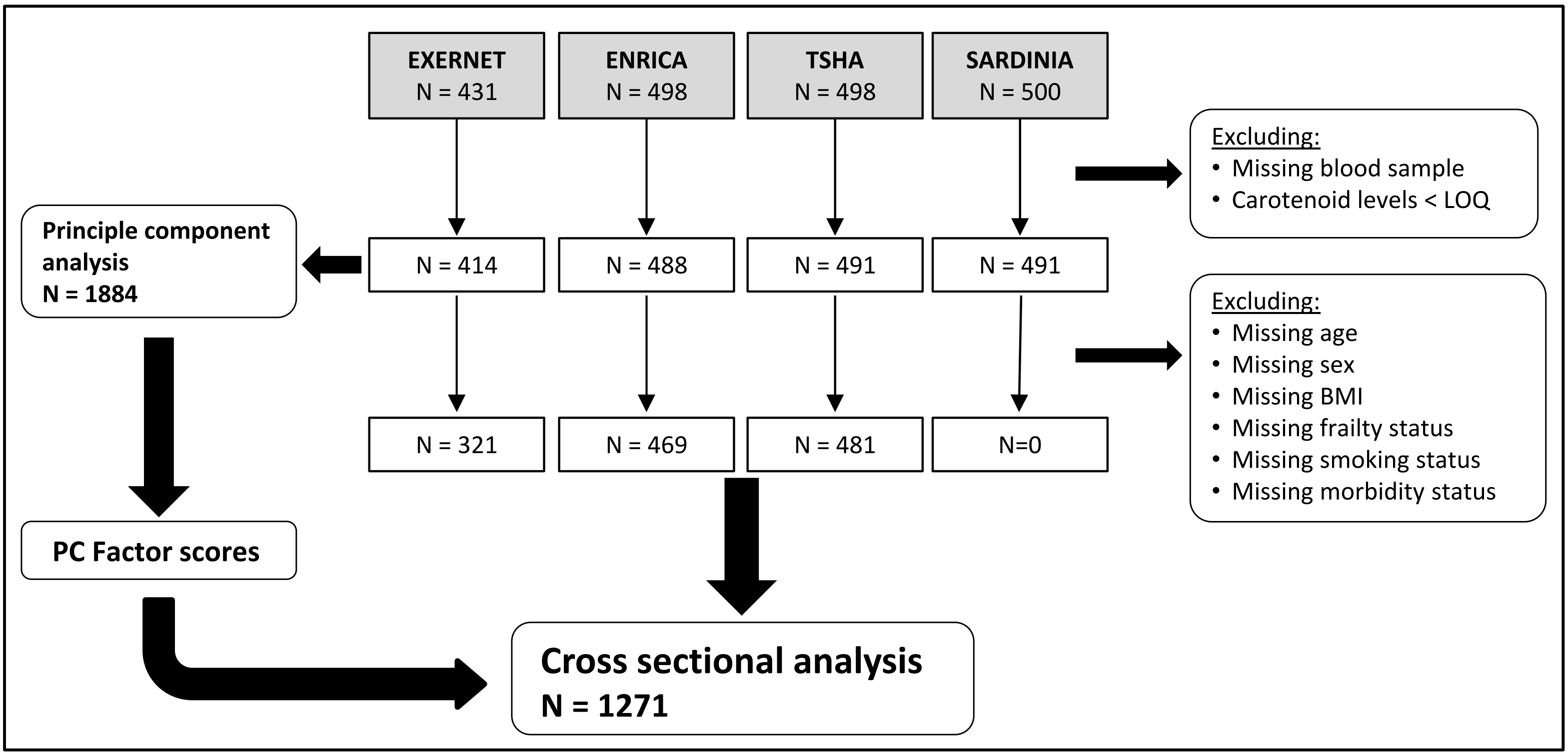

Supplemental Figure S1: Flow chart showing the exclusion criteria in dependency of the statistical analysis performed.
